# Development and Application of New Quality Model for Software Projects

**DOI:** 10.1155/2014/491246

**Published:** 2014-11-16

**Authors:** K. Karnavel, R. Dillibabu

**Affiliations:** Department of Industrial Engineering, Anna University, Chennai 25, India

## Abstract

The IT industry tries to employ a number of models to identify the defects in the construction of software projects. In this paper, we present COQUALMO and its limitations and aim to increase the quality without increasing the cost and time. The computation time, cost, and effort to predict the residual defects are very high; this was overcome by developing an appropriate new quality model named the software testing defect corrective model (STDCM). The STDCM was used to estimate the number of remaining residual defects in the software product; a few assumptions and the detailed steps of the STDCM are highlighted. The application of the STDCM is explored in software projects. The implementation of the model is validated using statistical inference, which shows there is a significant improvement in the quality of the software projects.

## 1. Introduction

**Figure 1:**
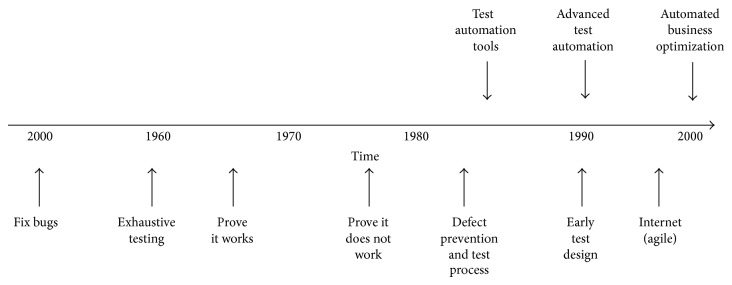
History of software testing.

Due to the rapid development of the software industry today, software companies are now facing a highly competitive market. To succeed, companies have to make efficient project plans to reduce the cost of software construction [1]. However, in medium to large scale projects, the problem of project planning is very complex and challenging [2, 3]. Despite numerous efforts in the past decade at defining and measuring software quality, the management of software quality remains in its infancy and the quality concept is still unutilized. The objectives of software testing are to qualify a software program's quality by measuring its attributes and capabilities against expectations and applicable standards. Software testing also provides valuable information to the software development effort. Throughout the history of software development, there have been many definitions and advances in software testing.  Figure 1 graphically illustrates these evolutions [4].

The life cycle development or waterfall approach breaks the development cycle down into discrete phases, each with a rigid sequential beginning and end. Each phase is fully completed before the next is started. Once a phase is completed, in theory during development, one never goes back to change it [4]. A software product is successful, if the requirements are fulfilled and no budget or deadline overflows occur [5]. So, the Software size contains important information for project planning. The method estimates the software size by counting the “function points” of the system. This method, developed by [6], is widely used in the USA and Europe.

Software quality assurance activities play an important role in producing high quality software. The majority of quality assurance research is focused on defect prediction models that identify defect-prone modules [7–11]. Although such models can be useful in some contexts, they also have their drawbacks. The literature makes two things clear about defect prediction. First, no single prediction technique dominates [12] and, second, making sense of the many prediction results is hampered by the use of different data sets, data preprocessing, validation schemes, and performance statistics [12–15]. These differences are compounded by the lack of any agreed reporting procedure or even the need to share code and algorithms [16].

**Figure 2:**
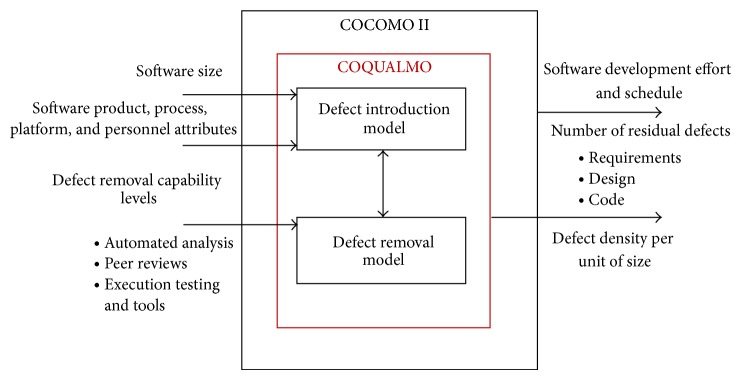
COQUALMO overview.

The COQUALMO model can play an important role in facilitating the balance of cost/schedule and quality [17]. Recognizing this important association, COQUALMO was created as an extension of the Constructive Cost Model (COCOMO), for predicting the number of residual defects in a software product. The COQUALMO model contains two submodels (the model shown in Figure 2):

1. the defect introduction model,
2. the defect removal model.

The defect introduction model uses a subset of COCOMO cost drivers and three internal baseline defect rates (requirements, design, code, and test baselines) to produce a prediction of defects, which will be introduced in each defect category during software development. The defect removal model uses the three defect removal profile levels, along with the prediction produced by the defect introduction model, to produce an estimate of the number of defects that will be removed from each category [17]. However, there are also a number of studies that do not confirm these results, especially regarding residual defects.

The importance of finding residual defects and reducing them (defect-free) in software products need not be accentuated. To reduce the number of defects during the design and development stage, well known product development tools such as the failure mode and effects analysis (FMEA), failure mode effects and criticality analysis (FMECA), quality function deployment (QFD), and sneak analysis (SA) are employed [18]. They provide the reader with a historical sketch of software testing, followed by a description of how to transform requirements to test cases, when there are well-defined or not so well-defined requirements.

This paper aims to overcome these issues and combine these models (waterfall and COQUALMO) to develop a suitable new model, namely, software testing defect corrective model (STDCM). The STDCM will serve as a better model for finding the number of defects and producing high quality software in the IT industry, without increasing the cost and schedule.

## 2. Limitations of COQUALMO

- The effort to fix the defect introduced and removed is not quantified directly by the model.
- COQUALMO does not associate the time aspect with the defect removal and fixing process.
- Defects are not given weights and classifications, in terms of the software artifact they originated from.

## 3. Motivation for Construction of Software Testing Defect Corrective Model

1. Can the existing model (waterfall and COQUALMO) achieve the maximum quality?
2. Is the prediction of errors necessary in the testing phase?
3. What are the possible ways to achieve the maximum quality of the model without increasing the cost and time?

**Figure 3:**
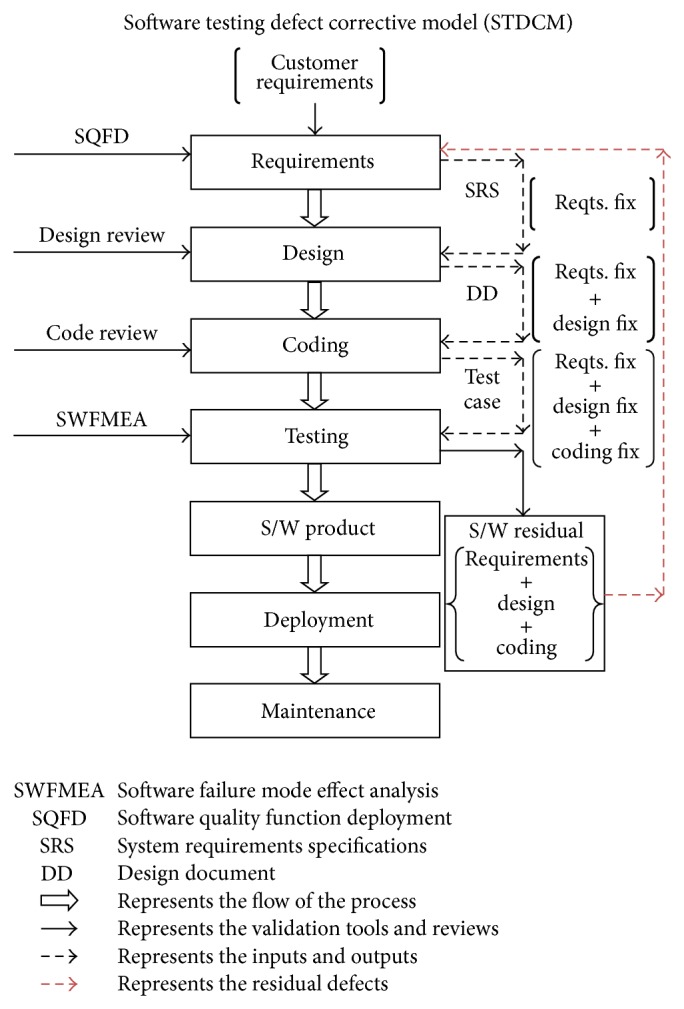
Framework of the software testing defect corrective model (STDCM).

These questions were answered by collecting the relevant literature in the area of software quality and a detailed Integrated model was constructed, as shown in Figure 3.

## 4. Related Works

This study was aimed at improving the quality and testing the process in the software projects, by reducing and finding the residual defects in software application projects. More numbers of papers relevant to the area are listed in below.

Phan et al. (1995) used IBM's OS/400 R.1 project to address key quality management and control issues in large development projects. The major task is improving the software quality during and after development. During the development of OS/400 R.1 at IBM corporation, thousands of programmers were involved in writing and refining millions of lines of code. Such an effort would fail without good software quality management. Hence, software developers cannot make good software quality products [19].

Cai (1998) applied a new static model for estimating the number of remaining defects and used a set of real data to test the new model. The new model resembles the Mills model in a particular case and is attractive in its applicability to a broader scope of circumstances. A practical example shows that the new model can offer good estimates for a number of software defects. It is also applicable to statistical problems other than software reliability modeling. They have not given a systematic review and hence cannot be applied for estimating the number of remaining defects [20].

Biffl et al. (2003) compared and investigated the performance of objective and subjective defect content estimation techniques. For the validation of these techniques they conducted a controlled experiment with 31 inspection teams, each of which consisted of 4–6 persons. They reported the data from an experiment with number of software engineering defects in a requirement document. They used the relative error, a confidence level, and the correctness for deciding reinspection as the main evaluation criterion, but they did not provide the major defects in the requirement document [21].

Chun (2006) applied a new method (capture-recapture model) that estimates the number of undetected errors in complex software design documents. This idea used the correlation matrix of multiple inspectors to formulate the estimation problem as a goal program. The capture-recapture model initially used by biologists to estimate the size of wildlife population has been widely used to estimate the number of software design errors. It shows that undetected errors are present and this leads to Software fault or failure [22].

Ravishanker et al.  (2008) applied the nonhomogeneous Poisson process model and the multivariate model, that applies Markov switching to characterize the software defect discovery process. However, the process remains complex and hence there is an increase in failure rate [23].

Jacobs et al.  (2007) studied the defect injection (DI) and defect detection (DD) influencing factors and their grouping, resulting in their use in development projects. To decrease the number of injected defects in a development project, the DI factors could be used as areas of attention, while the quality of documentation is poor, leading to a lack of product quality [24].

Quah et al. (2009) studied defect tracking as a proxy method to predict software readiness. They developed the defect predictive model, divided into three parts: (1) prediction model for presenting the logic tire, (2) prediction model for the business tier, and (3) prediction model for the data access tier. Evaluating the software readiness is very complex [25].

Chulani (1999) applied the COCQUALMO model to predict the defect density of the software under development, where defects conceptually flow into a holding tank through various defect introduction pipes and are removed through various defect removal pipes. In this model, it is difficult to increase the quality without increasing the cost and time. They inject the defects and remove them, but it involves more computation time, cost, and man power to predict the residual defects.

Westland (2004) analysed that the short software development life cycle appears to be in favor of higher rates of detection, but for some reasonable development cycle, most of the errors will be found incorrected. Short life cycles are likely to force constrained software development cycles and are likely to exacerbate the risk from postrelease defects. Defining uncorrected defects becomes exponentially costlier in each phase [26].

Turakhia et al.  (2006) used statistical testing to isolate the embedded outlier population, test conditions, and test application support for the statistical testing framework and the data modeling for identifying the outliers. The identification of outliers that correlate to latent defects critically depends on the choice of the test response and the statistical model's effectiveness in estimating the healthy-die response, but it provides low efficiency and less reliability, and the cost is very high [27].

Xiaode et al.  (2009) studied the quality prediction model and found that the number of faults is negatively correlated with the workload fluctuation, which indicates that the quality is decreasing due to the heavy workload. Due to the problems that occurred in the testing pahse for overheads, there is a decrease in the quality of the product [28].

Catal (2011) studied the software engineering discipline that contains several prediction approaches such as test effort prediction, correction cost prediction, fault prediction, reusability prediction, security prediction, effort prediction, and quality prediction. They investigated 90 software fault prediction papers published between 1990 and 2009. They gave a road map for research scholars in the area of software fault prediction [29].

However, the related works summarized are found to be more relevant for defect-free software products. “Estimating the remaining defects” and “predicting the residual defects” were found to be more suitable for the construction of STDCM. “Software estimation models” COQUALMO can play an important role in facilitating the balance of cost/schedule and quality. COQUALMO was created as an extension of the constructive cost model (COCOMO). The COQUALMO model contains two submodels.

The following conclusions can be drawn from the review of the literature.

- None of the alternatives is better than the others in all aspects.
- The waterfall and COQUALMO models do not emphasize more the correctiveness of software testing.
- The strengths and weaknesses of the other techniques are complementary.

## 5. Model Development Methodology

The STDCM has been developed based on two important traditional models, namely, waterfall and COQUALMO. The integration of these models was developed in a stagewise manner. The validation process of the model was built in a stagewise fashion. The framework of STDCM is shown in Figure 3. The following are the steps for the development of STDCM methodology.

**Step 1 (requirement analysis).**

The customer requirements of the project are collected in the requirements phase of STDCM. The requirements are analyzed using soft QFD (SQFD), in which the house of quality (HoQ) matrix was used, to validate the requirements of the given project.

**Step 2 (design).**

The input of this phase is SRS and the output is DD. This phase is validated using the design document process.

**Step 3 (coding).**

The lines of code are estimated using COCOMO/FPA. The code review process is used and it can be validated using statistical tools.

**Step 4 (testing).**

The inputs for this phase are the test cases and SWFMEA.

## 6. Application and Implementation of STDCM

A case study was conducted in a leading software company in India. The company was CMM level-5 certified in developing software projects for banking applications. It uses the offline review process for software quality improvement.

This project is basically used for credit card transaction (CCT) in the banking sector. It helps in the following cases:

- within project team communication validation,
- to address the credit card business problem,
- within project team URL (uniform resource locator) validation,
- centralized location for all URL and phone numbers,
- transaction services (TS) team with online screens to monitor and validate,
- a monthly scan process that allows for a continual review and update of the phone number and URLs.

The STDCM model was applied in this credit card transaction to improve the quality of project delivery. The following are the implementation details.

**Figure 4:**
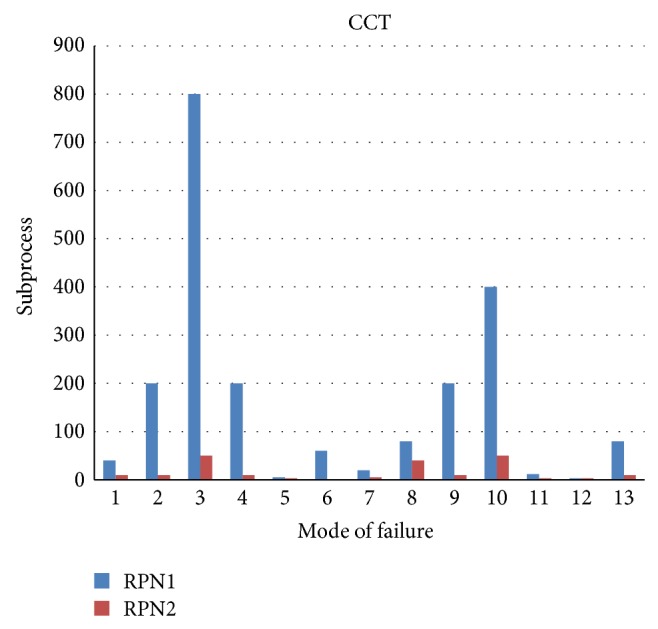
Bar chart for the RPN values (RPN 1: before implementing corrective actions; RPN 2: after implementing corrective action).

**Figure 5:**
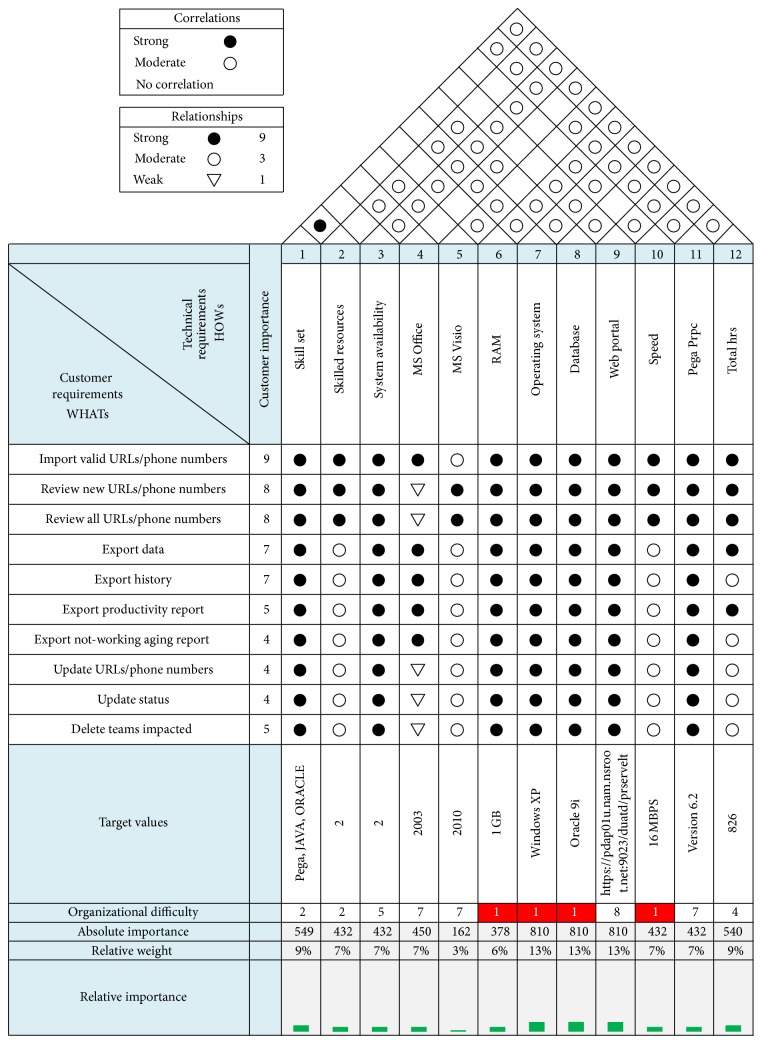
Software quality deployment diagram for finding correlations and relationships of customer requirements and functional requirements.

- The house of quality (HoQ) matrix was used to validate the correlations and relationships of customer requirements and functional requirements (see Figure 5).
- The absolute and relative importance of TRs are computed using the customer importance of CIs and the relationship ratings of the project (see Figure 5).
- The STDCM model conducted the code review process for the code developer and external code reviewer to reveal the code defects and resolve it (see Tables 1 and 2).
- The STDCM model includes occurrence rating, severity rating, and detecting ability rating table for finding the defects criteria (see Table 3).
- The STDCM models were implemented corrective actions in the developing software projects with the help of SWFMEA (see Table 4 and Figure 3).
- The SWFMEA is validated using the single paired *t*-test. The statistical paired *t*-test shows that there is significant improvement in the quality, in terms of the revised RPN values (Figure 4), due to the implementation of the SWFMEA (see Tables 5, 6, 7, and 8).
- A Paired correlation report highly correlated in the projects is 0.77. So there is a significant difference between RPN1 and RPN2. It increases the confidence level up to 98% (see Tables 6–8).

### 6.1. Building the Software-HOQ (Table 1)

#### 6.1.1. WHATs

Customer requirements are structured using affinity and tree diagrams and incorporated as WHATs of the software-HOQ. The WHATs are ranked in the order of importance using the analytic hierarchy process and comparison by pairs [30], which are shown in Figure 5. The priority of customer importance displayed in the HOQ next to each customer's voice has been obtained from the QFD team in a scale of 1 to 10. This gives the customer importance or priority rating for the WHATs of the software-HOQ. Number one implies low importance of priority and ten implies high importance of priority.

#### 6.1.2. HOWs

The HOWs usually represent the product features, design requirements, product characteristics, product criteria, substitutes of quality characteristics, and technical requirements. The HOWs represent the means by which a company responds to what the user wants and needs. These technical requirements are listed along the top of the software house of quality. Each technical requirement may affect one or more of the customer voices. Using the voice of the engineer table (Figure 5), the technical requirements were identified as similar to the customer requirements and are represented as HOWs in the software-HOQ.

The entire soft QFD process has been carried out by a QFD team with members from all departments (development, quality management, marketing, sales, service, etc.) and extended in several team meetings by the representatives of the client company.

### 6.2. Absolute and Relative Importance

#### 6.2.1. Absolute Importance

In Soft QFD applications, a cell (*i*, *j*) in the relationship matrix of HOQ, that is, *i*th row and *j*th column of HOQ, is assigned 9, 3, and 1 to represent a strong, medium, or weak relationship, respectively, between the *i*th customer requirement (CR) and the *j*th Technical requirement (TR). The absolute and relative importance of TRs are computed using the customer importance of CIs and the relationship ratings, that is, 9–3–1. For each TR, the absolute importance rating is computed as

(1) $$\begin{array}{c} {\text{AI}}_{i}=\overset{m}{\underset{i=1}{\sum }}{\text{CI}}_{i}*R_{ij}, \end{array}$$

where AI_*j*_ is the absolute importance of TR_*j*_, *j* = 1,…, *n*. CI_*i*_ is the customer importance, that is, importance rating of CR_*i*_, *i* = 1,…, *m*. *R*_*ij*_ is the relationship rating representing the strength of the relationship between CR_*i*_ and TR_*j*_. (*m*) Relative importance: the absolute importance rating can then be transformed into the relative importance rating, RI_*j*_, as

(2) RIj=AIjn,

(3) ∑k=1nAIk.

The larger the RI_*j*_ is, the more important the TR_*j*_ is. Thus, without consideration of any other constraints such as cost and time, TRs should be incorporated into the product of interest in the order of their relative importance rating to achieve more customer satisfaction.

The absolute importance AI_*j*_ for each technical requirement is calculated using (1). Referring to Table 1, the technical requirement “Skillset” has a strong relationship with the customer requirement “Import valid URLs/Phone Numbers.” Thus the column weight for the first column is computed as (9 × 9) + (8 × 9) + (8 × 9) + (7 × 9) + (7 × 9) + (5 × 9) + (4 × 9) + (4 × 9) + (4 × 9) + (5 × 9) = 549. The column weights are used to identify the technical requirements for quality improvement. The relative importance (RI_*j*_) for each technical requirement is calculated using (2).

### 6.3. Test of Hypotheses

Hypothesis testing helps to decide the basis of sample data, whether a hypothesis about the population is likely to be true or false. Statisticians have developed several tests of hypotheses (also known as tests of significance) for the purpose of testing of hypotheses which can be classified as (a) parametric tests or standard tests of hypotheses and (b) nonparametric tests or distribution-free test of hypotheses. Parametric tests usually assume certain properties of the parent population from which we draw samples. So, we have chosen parametric tests for testing our project samples [31].

#### 6.3.1. Important Parametric Tests

The important parametric tests are (1)  *z*-test, (2)  *t*-test, (3)  *X* ^2^-test, and (4)  *F*-test. All these tests are based on the assumption of normality; that is, the source of data is considered to be normally distributed. Our project sample data size is small; so, we have chosen the *t*-test. The relevant test statistic, *t*, is calculated from the sample data and then compared with its probable value based on *t*-distribution (to be read from the table that gives probable values of *t* for different levels of significance for different degrees of freedom) at a specified level of significance concerning the degrees of freedom for accepting or rejecting the null hypothesis [31].

#### 6.3.2. Hypothesis Testing of Means

Hypothesis testing refers to the formal procedures used by statisticians to accept or reject statistical hypotheses.

In such a situation *t*-test is used and the test statistic *t* is worked out as follows under

(4) t=X−μH0σs/nwith  df=n−1,σs=∑Xi−X−2n−1,

where $\overset{-}{X}$ = mean of the sample, *μ*_*H*_0__ = hypothesized mean for population, *σ*_*s*_ = standard deviation of sample, and *n* = number of items in a sample.

## 7. Conclusion

This paper discusses the development of a new model, namely, the STDCM, which uses the estimation of the COQUALMO and waterfall models. Its construction and applicability have been discussed. The limitations of other models are highlighted, in particular, the COQUALMO and waterfall. The framework shows the application of the SQFD and SWFMEA in STDCM and the size estimation computation of COCOMO/FPA. The STDCM will serve as a better model for finding the number of defects and producing high quality software in the software industry. The model was demonstrated using banking applications and specializes in developing credit cards. Moreover, the model was successfully validated, using the statistical inference.

## Figures and Tables

**Table 1:** Code review form.

**Project name:** Credit Card Transaction Code Review Form											
**Date of walkthrough:** **10/11/2011**											

Sl.no.	Issue raised by	Issue owner	Open date	Status (open/closed)	Severity S, H, M, L	Code language	Impacted module	Issue description	Was module unit tested (yes/no)	Resolution	Closed date
1	XXX	YYY	10/11/2011	Closed	L	PEGA	CCT delete screen	While using embedded pages in the activities like CCT DeleteRow, instead of using the following when condition ContactsPage.pxResults (local.i).pyRowSelected==“true”	Yes	.pyRowSelected==“true”	10/12/2012

2	XXX	YYY	10/11/2011	Closed	L	PEGA	CCT update screen	When step page is pyWorkPage instead of using the following when condition pyWorkPage.URL_NUMBER ==pyWorkPage.URL_NUMBER_ NEW&&pyWorkPage.ChoiceCCT HomeScreen==“Update”	Yes	We can prefer using .URL_NUMBER==.URL_ NUMBER_NEW&&.Choice CCTHomeScreen==“Update”	10/12/2012

3	XXX	YYY	10/11/2011	Closed	L	PEGA	CCT review all screens	Steps with many labels	Yes	Step description can be given to avoid confusion	10/12/2012

**Table 2:** Requirements and application issue count reports for the CCT project.

Application/project	Credit card transaction (CCT)
Requirements	(1) Improve valid URLs/phone numbers in the database
	(2) Review new URLs/phone numbers
	(3) Review all URLs/phone numbers
	(4) Export the history/entire data
	(5) Export not-working aging report and productivity report
	(6) Update URLs/phone numbers
	(7) Update status
	(8) Delete teams impacted
Rules/LOC	776
Estimation splitup (hrs)	780 + 46
Total estimation (hrs)	826
Code issue count	12
Data issue count	22
Application issue count	2

**Table 3:** Occurrence rating process SWFMEA.

Occurrence rating		
Probability of failure	Rating	Criteria
Very frequently	**10**	Shows greater than 80% of defects attributable to subprocess failure
Frequently	**8**	Shows greater than 40% of defects & less than 80% of defects attributable to subprocess failure
Sometimes	**4**	Shows greater than 10% of defects & less than 40% of defects attributable to subprocess failure
Rare	**1**	Shows less than 10% of defects to subprocess failure.

Severity rating		
Effect	Rating	Criteria

Very high	**10**	Rework is greater than 8 ph
High	**7**	Rework is greater than 4 ph & <8 ph
Moderate	**5**	Rework is greater than 2 ph & <4 ph
Low	**3**	Rework is greater than 1 ph & <2 ph
Very low	**1**	Rework is greater than 1 ph

Detectability rating		
Detection	Rating	Criteria

Difficult	**10**	No defined methods for identifying the process error; only the o/p prdt analysis leads to detectability
Moderate	**5**	Can be identified in the exit phase of the process or subsequent process entry check
Easy	**1**	Process has built in checks for identifying subprocess failure

**Table 4:** Complete processes of software failure mode and effect analysis.

CCT																
Function/process	Subprocess	Mode of failure	Effect of failure	Cause of failure	Current controls	Current status				Recommended corrective actions	Action by	Action taken	Revised status			
						OCC	SEV	DET	RPN1				OCC	SEV	DET	RPN2
Coding	Planning	Inadequate skills/knowledge about the process	Unable to access the application	Request has not been raised to get access	This should be added in implementation checklist	4	10	1	40	Assigning SPOC to get access	Project manager (PM)	Knowledge transition about process	1	10	1	10

Coding	Planning	Inadequate time	Delivery not in time	Incorrect estimated hours	Estimation should be done by skilled resources	4	10	5	200	Project owner/lead has to review the estimation based on requirements given	Project manager (PM)	To follow estimation model with proper training & knowledge	1	10	1	10
		Requirement change				8	10	10	800				1	10	5	50
		Inappropriate skilled resources for DEV				4	10	5	200				1	10	1	10
		Testing & review objective is not clear				1	5	1	5				1	3	1	3
		Inappropriate skilled resources for testing & review				4	3	5	60				1	1	1	1

Coding	Development	Not reusing the existing rules	File size is huge to move	Increase in the count of rules	Code review should be done to check the logic/rules handled	4	5	1	20	Proper training and development	Project manager (PM)	Review by skilled resources	1	5	1	5

Coding	Testing	Invalid data	Defects raised	Improper data handled while testing	Proper unit test scripts should be provided	8	10	1	80	Proper training about the application	Project manager (PM)	Valid unit test script template is followed with valid values & validations	4	10	1	40
		Testing objective is not clear				4	10	5	200				1	10	1	10
		Inappropriate skilled resources for testing				4	10	10	400				1	10	5	50

Coding	Review	Inappropriate skilled resources for review	Defects raised	Reviewed unnecessary codes	Should give the details about the code to be reviewed	4	3	1	12	Sharing the code details with required rule set & rule set version	Project manager (PM)	To follow IQA, EQA, and FI process before implementation	1	3	1	3
		Inadequate time				1	3	1	3				1	3	1	3
		Improper details about code review				8	10	1	80				1	10	1	10

**Table 5:** SWFMEA validation report (RPN1 TO RPN2).

CCT						
H_0_: not significant in RPN1 & RPN2						
H_1_: significant in RPN1 & RPN2	*N* = 13, SQRT(*N*) = 3.60551					

Mode of failure	RPN1	(*x* − *μ*)	(*x* − *μ*)²	RPN2	(*x* − *μ*)	(*x* − *μ*)²

Inadequate skills/knowledge about the process	40	−121.538	14771.6	10	−5.76923	33.28402
Inadequate time	200	38.46154	1479.29	10	−5.76923	33.28402
Requirement change	800	638.4615	407633.1	50	34.23077	1171.746
Inappropriate skilled resources for DEV	200	38.46154	1479.29	10	−5.76923	33.28402
Testing & review objective is not clear	5	−156.538	24504.29	3	−12.7692	163.0533
Inappropriate skilled resources for testing & review	60	−101.538	10310.06	1	−14.7692	218.1302
Not reusing the existing rules	20	−141.538	20033.14	5	−10.7692	115.9763
Invalid data	80	−81.5385	6648.521	40	24.23077	587.1302
Testing objective is not clear	200	38.46154	1479.29	10	−5.76923	33.28402
Inappropriate skilled resources for testing	400	238.4615	56863.91	50	34.23077	1171.746
Inappropriate skilled resources for review	12	−149.538	22361.75	3	−12.7692	163.0533
Inadequate time	3	−158.538	25134.44	3	−12.7692	163.0533
Improper details about code review	80	−81.5385	6648.521	10	−5.76923	33.28402

Total	2100		599347.2	205		3920.31
Mean (*μ*)	161.5385		49945.6	15.76923		326.6925
Standard deviation (*σ*)	223.4851			18.07464		
Variance (*σ* ^2^)	49945.6			326.6925		
Standard error mean (*σ*/SQRT(*N*))	61.98433			5.01306		

H_0_: there is no significant difference between RPN1 and RPN2.

H_1_: there is a significant difference between RPN1 and RPN2.

**Table 6:** Paired correlations report.

To find correlations (2 tailed)		
Correlation (*r*)	0.773723	
*P* value	0.027661	<0.05 (so reject H_0_)

0.77: it is highly correlated. So there is a significant relationship between RPN1 and RPN2.

**Table 7:** Paired samples test.

Paired samples test (2 tailed)		
Paired difference mean	145.7692	
Paired difference standard deviation (*σ*)	205.4105	
Paired difference standard error mean (*σ*/SQRT(*N*))	56.97127	
*σ* ^2^/(*N* − 1)	4162.134	
Total *σ* ^2^/(*N* − 1)	4189.358	
SQRT(*σ* ^2^/(*N* − 1))	64.72525	
*t* = Paired Difference Mean/SQRT(*σ* ^2^/(*N* − 1))	2.252123	
Degrees of freedom (df)	12	
*t* table value	1.78	**<2.25 (so reject ** **H**_0_ **)**

**Table 8:** Confidence limits reports.

To find confidence limits (2 tailed)	
*t*(0.025,12)	2.18
Lower level confidence limit	21.57186
Upper level confidence limit	269.9666

Confidence level: 98% ± 5% accuracy.
